# Clinical determinants associated with quality of life for people who live with HIV/AIDS: a Meta-analysis

**DOI:** 10.1186/s12913-019-4659-z

**Published:** 2019-10-29

**Authors:** Hesam Ghiasvand, Katherine M. Waye, Mehdi Noroozi, Gholamreza Ghaedamini Harouni, Bahram Armoon, Azadeh Bayani

**Affiliations:** 1 Social Determinants of Health Research Center, University of Social Welfare and Rehabilitation Sciences, Tehran, Iran; 2 Department of Epidemiology, Brown University School of Public Health, Providence, RI USA; 3 Social Welfare Management Research Center, University of Social Welfare and Rehabilitation Sciences, Tehran, Iran; 4 Social Determinants of Health Research Center, Saveh University of Medical Sciences, Saveh, Iran; 5 Social Determinants of Health Research Center, Yasuj University of Medical Sciences, Yasuj, Iran; 6 Student Research Committee, School of Allied Medical Sciences, Shahid Beheshti University of Medical Sciences, Tehran, Iran

**Keywords:** Clinical aspects, Quality of life, People who lived with HIV/AIDS infection, Meta- analysis

## Abstract

**Background:**

During recent years, Quality of Life (QoL) is a significant assessment factor in clinical trials and epidemiological researches due to the advent of Antiretroviral Therapy (ART), Human Immunodeficiency Virus (HIV) has become a manageable,chronic disease. With regards, more attention must be paid to the QoL of infected patients. Limited evidence exists on the impact of ART on QoL among HIV infected patients. Due to lacking of a systematic approach to summarizing the available evidence on the clinical determinants of People Who Live with HIV/AIDS (PWLHs’) QoL, this study aimed to analyze the impact of clinical determinants (ART experience, CD4 count < 200, co-morbidities, time diagnosis and accessibility to cares) on QoL among PWLHs’.

**Methods:**

This study was designed in accordance with Preferred Reporting Items for Systematic Reviews and Meta-Analyses (PRISMA). PubMed, Science Direct, Web of Science, and Cochrane electronic databases were searched in February 2017 to identify all past studies that discussed social and behavioral characteristics of QoL in PLWHA. To recognize effective factors on social and behavioral QoL, a meta-analysis was conducted. Polled Odds Ratios (ORs) were utilized at a 95% confidence level. Since sampling methods differed between articles in the systematic review, we evaluated pooled estimates using a random effect model. Metan, metareg, metacum, and metabias commands in STATA version 13.0 were applied to analyze the data**.**

**Results:**

Our findings indicated that ART has a positive impact on QoL, with a pooled effect size at approximately 1.04 with a confidence interval between 0.42 to 1.66 which indicates this impact is not very considerable and may be relatively neutral. The pooled effect size for CD4 count on QoL was .29 (95%CI = .22–.35), indicating that there is a negative associate between CD4 count and QoL. The co-morbidity as a negative determinant for QoL among HIV/AIDS infected people. The pooled effect size implies on a relative neutral association, although the confidence interval is wide and ranges between 0.32 to 1.58. The pooled effect size is about 1.82 with confidence interval 1.27 to 2.37 which indicates a considerable positive association with lowest level of heterogeneity.

**Conclusions:**

The results illustrated that time diagnosing and availability to hospital services had significant relationship with a higher QoL and CD4 < 200 was associated with a lower QoL. In conclusion, policy makers should set an agenda setting to provide a suitable diagnostic and therapeutic facilities to early detecting and continues monitoring the health status of People Who Live with HIV/AIDS (PWLHs’).

## Background

QoL has become a critical assessment factor in clinical trials and epidemiological research during the past years as researchers begin to acknowledge the impact of QoL has on overall wellbeing. QoL consists of four dimensions: mental and physical health, communication, and functional autonomy [1]. With the implementation of antiretroviral therapy (ART) as an effective prevention and treatment method, HIV has become a chronic disease if PWLHs’ adhere to their treatment. Therefore, it is imperative to understand the QoL for PWLHs’ as they continue to live with HIV and reach for viral suppression [2]. Research has shown that HIV affected patients not only suffer from physical issues, but also experience stigma and misjudgment from society, which can negatively impact an individual’s QoL [3].

More than 60 million people suffer from HIV which is one of the main causes of premature death in developing countries [4]. According to the United Nations joint program on HIV and AIDS (UNAIDS) [5], the incidence rate of HIV infection has reduced to 38% per year for example, the global HIV incidence rate was 3.4 million in 2001 and 2.1 million in 2013 [6]. In order to improve well-being and social and individual function of patients with HIV infection, critical elements of their QoL must be examined. In addition, recognition of negative factors that impact their QoL could be helpful in improving tailored health care services that enhance QoL gradually [7].

Some of the greatest factors the negatively effect the success of ART on PWLHs’ are adherence, substance abuse, and refusal in practicing safe sex [8]. On the other hand, studies have identified some positive factors that enhance QoL for PWLHs’, including physical and mental health, socio-demographic characteristics, social network, and job status [9–12]. Social and behavioral plans must be systematically integrated to potentially lower risk and consequently, disease transmission. However, there is a dearth in literature, specially with meta-analyses and comprehensive systematic reviews, regarding QoL of HIV infected patients receiving ART [13].

Studies have found that the life expectancy of HIV infected patients from high income nations that had received appropriate combination of antiretroviral treatment (cART), were equal to that individuals without HIV infection. Nonetheless, factors affecting health-related quality of life (HRQoL) are not separately examined between people with HIV infection and the general population [14].

Improvements in QoL for PWLHs’ reduce the risk for probable infections associated with HIV, and improves survival for those receiving ART. Positive possible measurements that can assess from ART clinical results are lower mortality rate, severe AIDS-related symptom, and a decrease in the rate of opportunistic infection [15]. ART has proven to be effective if adhered to and has the potential to improve QoL and the general health of patients. Numerous factors associated with an improved QoL among PWLHs’ are: greater co-morbidity burden [16] and AIDS detection [17] were unambiguously related to lower QoL through the lifetime. ART treatment influences the QoL [18]. Additional factors include lower HIV viral load [19], higher CD4+ cell count [19–21] and less symptoms associated with HIV [22]. There is limited evidence on the impact of prevention plans and QoL, further meta-analyses and comprehensive systematic reviews focusing QoL among PWLHs’ who receive ART are lacking from the literature [13]. Therefore, the aim of this meta-analysis is to summarize the available clinical evidence reports (CD4 count, ART etc..) and its effects on QoL. Due to lacking of a systematic approach to summarizing the available evidence on the clinical determinants of PWLHs’ QoL, this study aimed to analyze the impact of clinical determinants (ART experience, CD4 count < 200, co-morbidities, time diagnosis and accessibility to cares) on QoL among PWLHs’.

This review can provide direction to policy makers, public health planners and program developers on how best to use their resources to improve the QoLof HIV infected people**.**

### Study question

What are the impacts of the clinical determinants on QoL among PWLHs’?

## Methods

### Search strategy and study selection

This study was designed in accordance with identified characteristics from reports on the Preferred Reporting Items for Systematic Reviews and Meta-Analyses (PRISMA) [23–25].

To identify all published, peer-reviewed literature on the social and behavioral characteristics of QoL among PWLHs’, we (H.GH and B.A) searched PubMed, ScienceDirect, Web of Science, and Cochrane electronic databases independently from February 2000 to February 2017 (H.GH. and B.A.) (Table 1: represents the search strategy.)

**Table 1:** Search strategy

In [Title]	• #1 (Acquired immune deficiency syndrome [Title/Abstract] OR AIDS [Title/Abstract])
	• #2 (Human immunodeficiency Virus Infection [Title/Abstract] OR HIV Infection [Title/Abstract])
	• #3 (Quality of Life [Title/Abstract] OR QoL [Title/Abstract])
	• #1+ #2
	• #2+ #3
In [Title, Abstract, Keyword]	• #1 “Acquired immunodeficiency syndrome”: ti, ab,kw
	• #2 “AIDS”: ti,ab,kw • #3 “Human immunodeficiency Virus Infection”: ti, ab,kw
	• #4 “HIV infection”: ti, ab, kw
	• #5 “Quality of Life”: ti, ab, kw
	• #6 “QoL”: ti, ab, kw
	• #7 “#1+ #5”
	• #8 “#1+ #6”
	• #9 “#2+ #5”
	• #10 “#2+ #6”
	• #11 “#3 + #5”
	• #12 “#3 + #6”
	• #12 “#4+ #5”
	• #13 “#4 + #6”

### Inclusion criteria

Initial inclusion criteria were articles with at least an English abstract as well as articles of all times and geographic locations.. After reviewing for duplication, selected articles were assessed for accordance with title, abstract and relevance to the subject of study. Following PICO guidelines, additional inclusion criteria during secondary review were any individual living with HIV among any age group (population), Highly Active Antiretroviral Therapy (HAART) and ART interventions on QoL (intervention), participants that did not receive ART or HAART (comparison group), studies that reported HIV/AIDS clinical information (outcomes), and any study type (cross-sectional, cohort, and case-control) were included (studies). Qualitative studies, Meta-analysis, Systematic reviews and secondary studies were excluded. Also studies with published papers in any language except English were excluded as well.

### Data extraction and study quality assessment

Two independent reviewers (H.GH. and B.A.) investigated the titles and abstracts, in accordance with the inclusion and exclusion criteria of the study. Any disagreements between the reviewers were discussed by the two reviewers until consensus reached. A third person (E.A) from research team provided input as needed. Then, these reviewers reviewed the full-texts, observing the inclusion and exclusion criteria. Data were independently extracted by the researchers and catergorized based on article characteristics: first author, publication year, demographic data of sample (age, job, income, education) and other aspects like substance use, alcohol consumption, safe intercourse, and social support (Table 2). Newcastle-Ottawa Scale [26] suggested by the Cochrane Collaboration [27] was used to assess the quality of evaluated articles.

**Table 2:** Sample Description

Authors	Country	Year	Sample Size	Study Design	Instrument for measuring QoL	Quality Assessment Criteria
Aragonés-López et al.	Cuba	2012	354	Cross- sectional	None	Good
Deribew A. et al.	Ethiopia	2013	465	Cross-sectional	None	Satisfactory
George et al.	Ireland	2016	521	Cross- sectional	None	Good
Tesfaye et al.	Ethiopia	2015	250	Cross- sectional	WHOQOL^a^	Good
Rüütel K., et al	Estonia	2009	451	Cross- sectional	WHOQOL	Satisfactory
Boyer et al.	France	2012	1985	Cross- sectional	SF-12 physical (PCS)^b^ and mental (MCS) HRQL^c^	Good
Lan et al.	China	2016	261	Cross- sectional	MOS^d^	Good
Friend-du Preez et al.	South Africa	2009	612	Cross- sectional	WHOQOL	Good
Ruiz Perez et al.	Spain	2005	320	Cross- sectional	MOS	Satisfactory
Louwagie et al.	South Africa	2007	371	Cross- sectional	EQ5D^e^	Good
D Nglazi et al	South Africa	2014	903	Cross- sectional	EQ5D	Good
Briongos Figuero et al.	Spain	2011	150	Cross- sectional	MOS	Good

^a^WHOQOL

^b^PCS

^c^HRQL

^d^MOS

^e^EQ5D

### Data synthesis and statistical analysis

To discover effective social and behavioral factors on QoL, a Meta-analysis utilizing pooled odds ratios (ORs) and the 95% cconfidence intervals was conducted.

Dixon Q-test with a *P* value < 0.05 and I2 statistics with a cut off ≥50% was utilized to evaluate correlation status between the two researchers. 95% confidence intervals for I^2^ was assessed where negative values of 12 were considered zero. To evaluate pooled estimates, a random effect model was applied to adjusted for differeing sampling methods within the studies. Additionally, a subgroup analysis was conducted to determine heterogeneous articles.

A cumulative regression analysis was conducted, to estimate the trend of ORs over time. In order to recognize publication bias, we used Begg’s and Egger’s publication bias method in statistical and graphical modes [3, 28]. A *p*-value<.05 was considered statistically significant. Physical aspect of QoL and the effect of social and behavioral influences were explored using ORs and 95% CI that were displays on forest plots. Metan, metareg, metacum, and metabias commands in Stata version 13.0 were applied to analyze the data (Stata Corporation, College Station, TX).

## Results

### Study characteristics

**Fig. 1:**
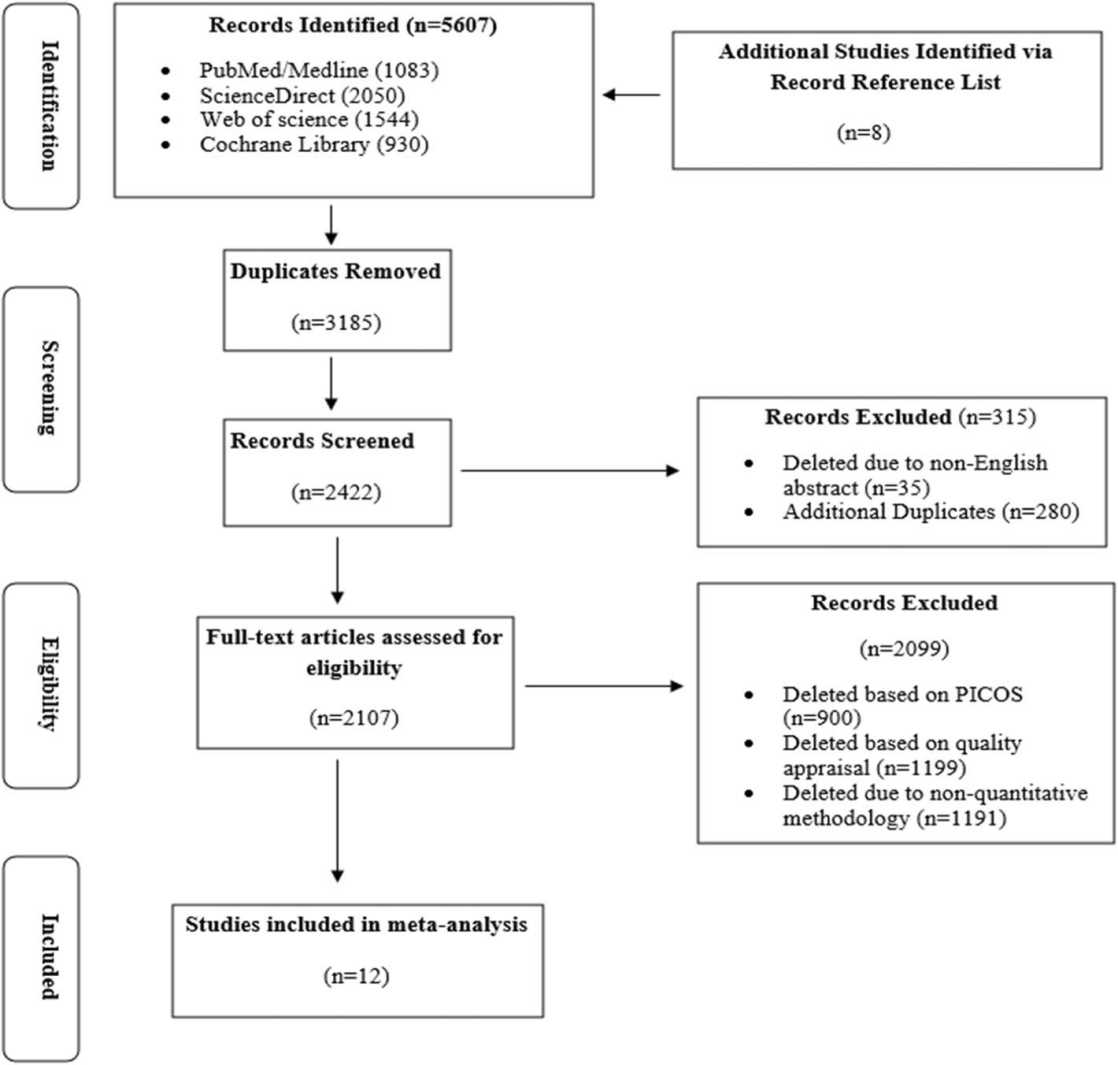
PRISMA study design

A flow diagram depicting the review process is presented in Fig. 1**.** In total, 5607 papers were identified from four databases and from searching reference lists. Of these, 2107 articles were selected for full text review. After detailed assessment of the citations, we included 12 studies [2, 4, 7, 29–37]**.** Table 2 depicts the chatracteristics of the studies.

### Results of metanalysis

**Fig. 2:**
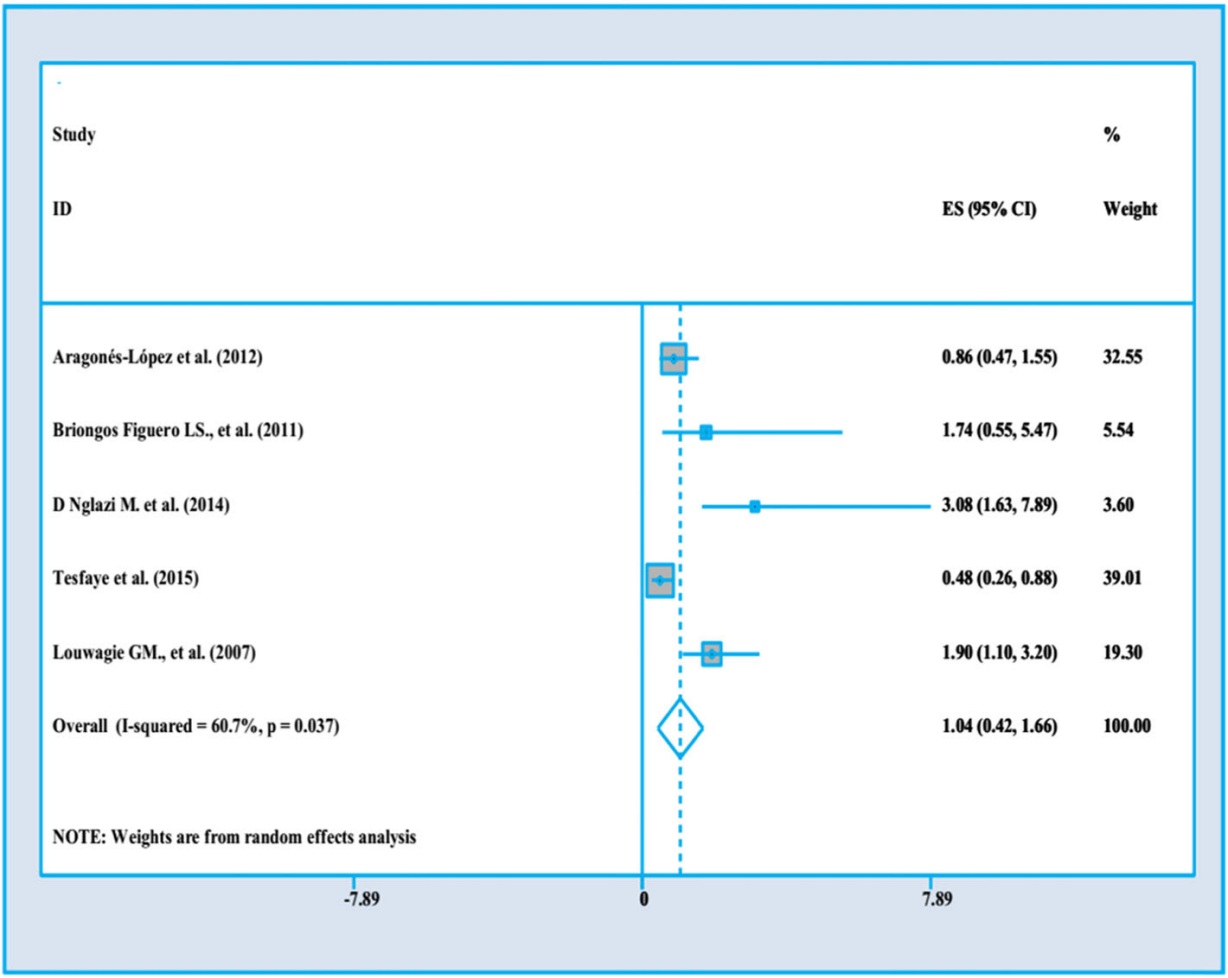
The impact of Anti-Retroviral Therapy on PLWHA’s QoL

There are several clinical factors influencing PWLHs’ QoL in studies. These are shown in Fig. 2 to Fig. 5.

### The impact of ART/HAART on PLWHA’s QoL

In our meta-analysis we considered 5 studies evaluating the impact of ART/HAART on PLWHA’s QoL. These studies discussed about the impact of ART/HAART as an exposure variable and PLWHA’s QoL as an outcome variable. These researches have been done between 2007 to 2015, the sample sizes were from 150 to 903 with a high quality structured approach.

Three studies were implemented in upper middle income country (such as South Africa and Cuba) [29, 35, 37] the study of Tesfaye et al. [32] was done in lower income country (Ethiopia) and the study of Briongos Figuero et al. [4] was done in higher income country (Spain). All of them considered studies used cross-sectional analysis and evaluated impact of ART as a current measure using self-reported questionnaire. As illustrated in Fig. 2**,** ART/HAART does not have any impact on QoL (OR = 1.04, 95%CI = .42–1.66). The overall heterogeneity was about 60.7%.

### The impact of CD4 count < 200 on PWLHA’s QoL

**Fig. 3:**
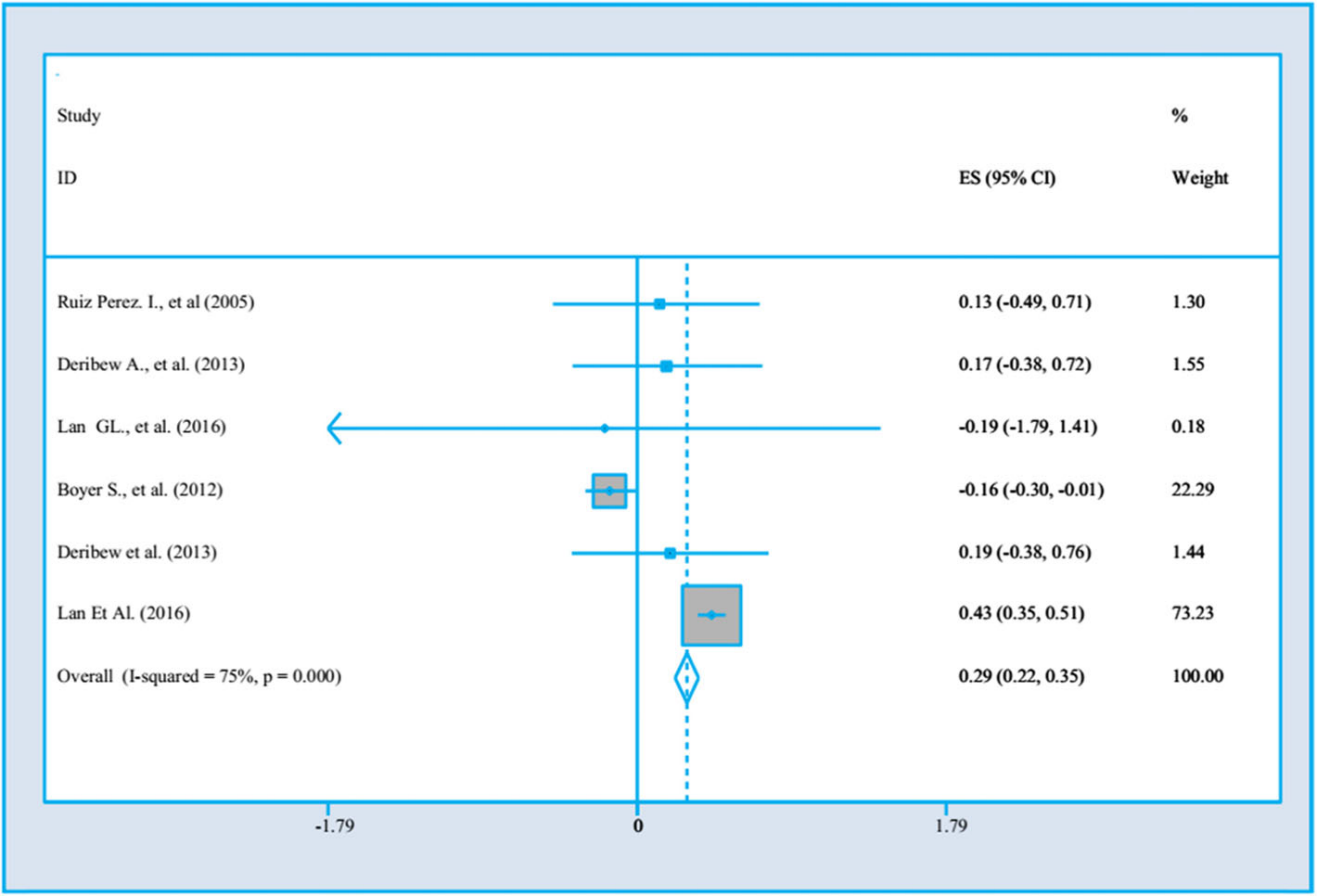
The impact of CD4 < 200 numbers on PWLHs’ QoL

Four studies [20, 30, 33, 34] examined the impact of CD4 count < 200 on PWLHA’s QoL. Two studies implemented in high-income countries [20, 33], one research was from a upper middle-income country [34] and the last study conducted from a low income country [30]. The date of studies were from 2005 to 2016, and the sample size ranged from 261 to 1985. Two studies had week quality [20, 30] approaches and other researches were categorized as the high quality [33, 34]. All of four studies used cross –sectional design. The progression of HIV/AIDS infection is presented through two indicators CD4 count and the recent World Health Organization’s HIV infection [38] staging approach. The results of CD4 count on PLWHA’s PWLHs’ QoL have been presented in Fig. 3**.** There is a reverse association between CD4 count (CD4 < 200) and QoL. Among PWLHs, those who had CD4 < 200 were 0.29 times less likely to have poor quality of life (OR = .29, 95%CI = .22, .35) and the heterogeneity is about 75%.

### The impact of co-morbidities on PLWHA’s QoL

**Fig. 4:**
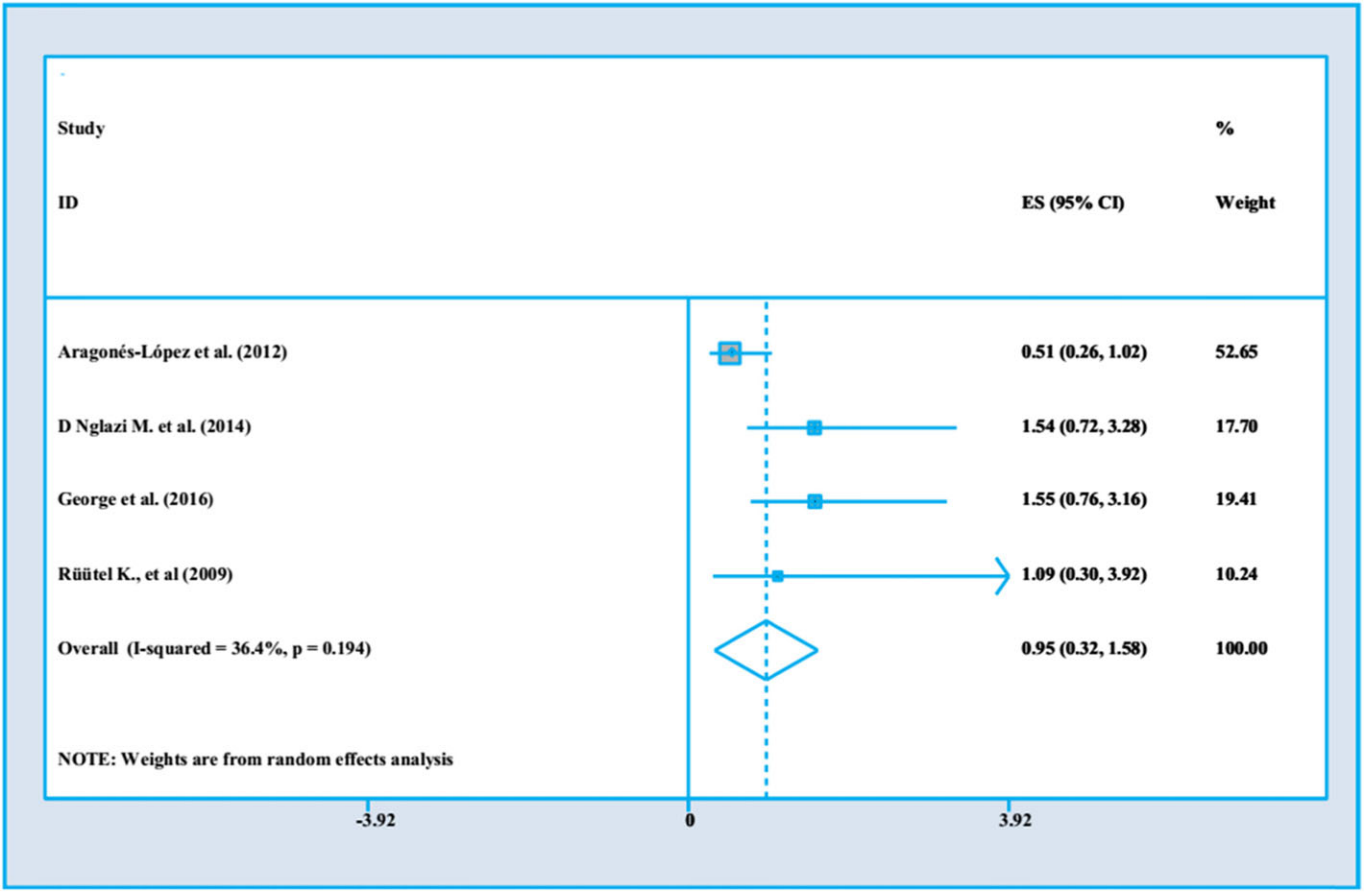
The impact of Co-morbidity on PWLHs’ QoL

Our finding indicated that four studies evaluated the impact of co-morbidities on PLWHA’s QoL. Of these two of them were done in upper midle income country [29, 37] and two were from higher income country [7, 31]. These studies were from 2009 to 2016, and the sample size ranged from 354 to 903. Three studies had high quality [29, 31, 37] approaches and one research were categorized as the week quality [7]. All of four studies used cross –sectional design. Figure 4 indicates that co-morbidities do not (HCV, HBV and Tuberculosis) have an effect on QoL among PWLHs’ (OR = .95, 95%CI = .32–1.58). The heterogeneity statistics is about 36.4%.

### The impact of in time diagnosis and accessibility to cares on PWLHs’ QoL

Our result demonstrated that four studies assessed the impact of co-morbidities on PLWHA’s QoL. Of those two of them were done in upper midle income country [29, 35] and two were from higher income country [4, 7]. These studies were from 2009 to 2012, and the sample size ranged from 150 to 612. Three studies had high quality [4, 29, 35] and one research were categorized as the week quality [7]. All of four studies used cross –sectional design.

**Fig. 5:**
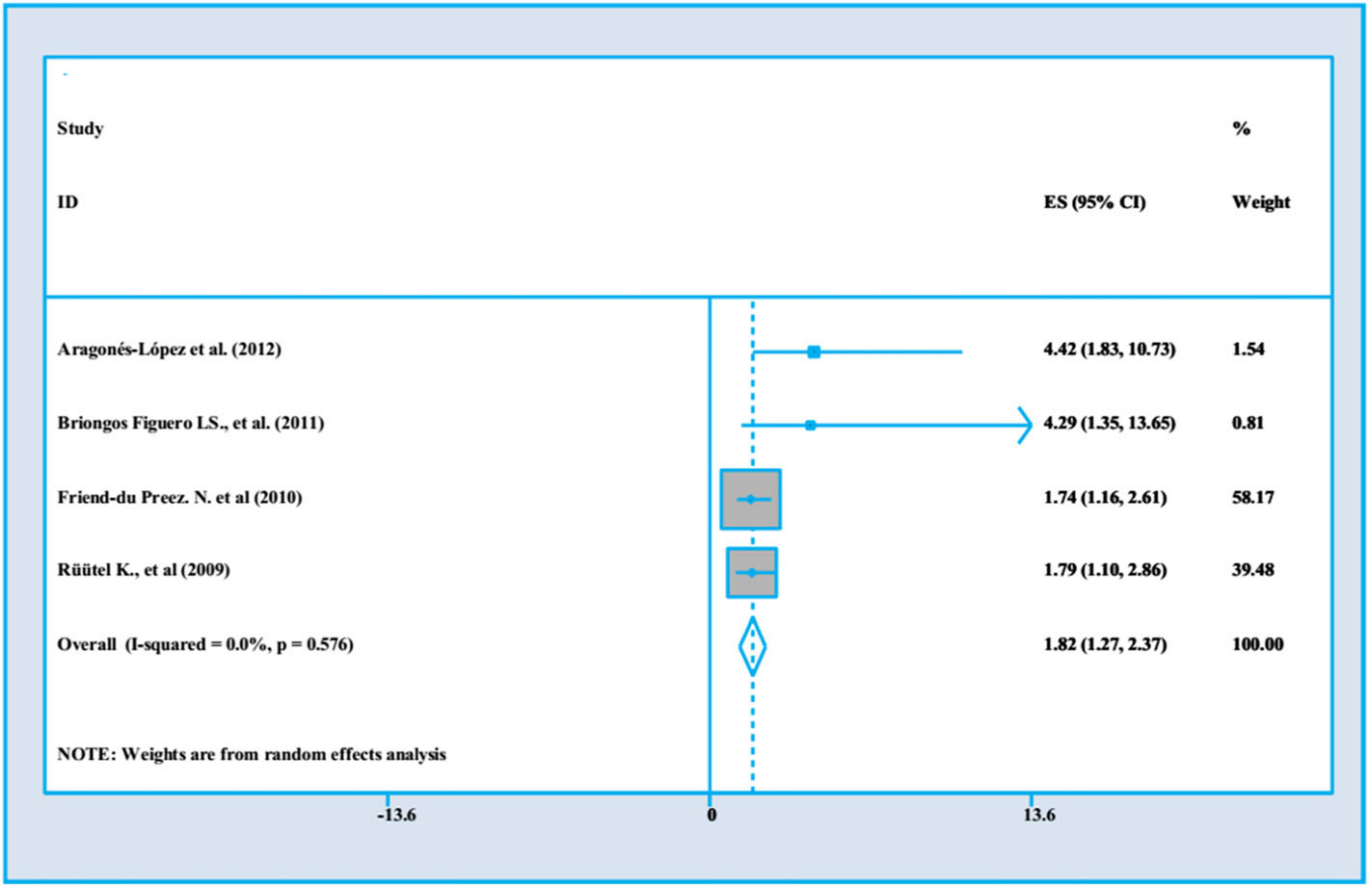
The impact of in time diagnosis and accessibility to cares on PWLHs’ QoL

Figure 5 depicts the association between in-time diagnosing and accessibility to hospital services on QoL in PWLHs’. In respondents those who have early time diagnosing were 1.82 times more likely to have accessibility to hospital services (OR = 1.82, 95%CI = 1.27, 2.37).

**Fig. 6:**
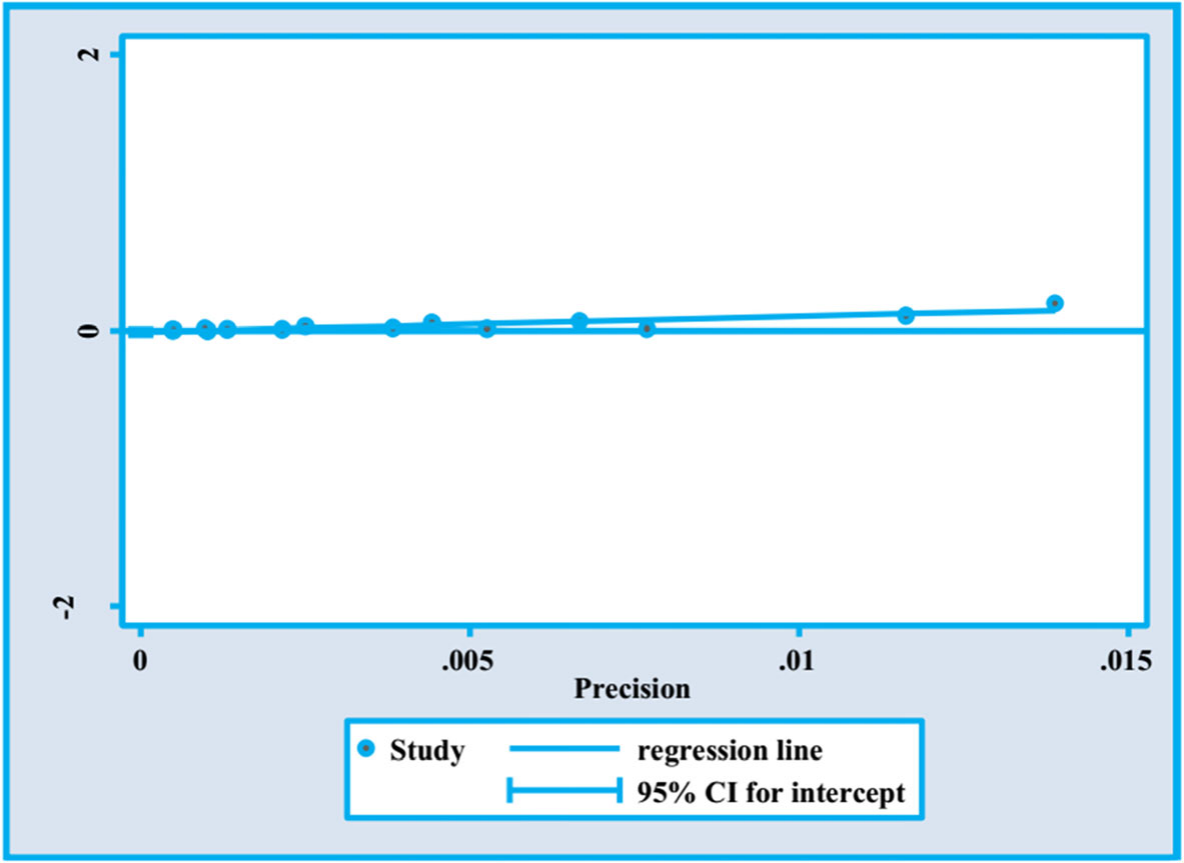
Publication bias using Egger’s plot

To identify publication bias, we utilized Egger’s plot which did not demonstrate significant bias (*p* = 0.31) (Fig. 6).

## Discussion

This study aimed to analyze the impact of clinical determinants (ART experience, CD4 count < 200, co-morbidities, time diagnosis and accessibility to cares) on QoL among PWLHs’.

It is important to note that ART/HAART was not associated with improved or lowered QoL in this meta-analysis. This may be attributable to the complexity of the therapeutic regimen as well as the number and doses of drugs taken daily. Basavaraj et al. in their review concluded that the development of ART could change the patients’ perception about the AIDS as a fatal disease to a manageable one [39]. However, some studies have highlighted that adherence to ART may be a more effective determinant on PWLHs’ QoL rather than uptake of ART. Adherence entails guaranteeing the availability and accessibility to therapy alongside effective counseling techniques to encourage those utilizing ART to adhere to treatment [40, 41].

The influence of ART on HRQoL has been introduced as a equilibrium between decreased HIV-related symptoms and enhanced life-expectancy in an aspect and medication side-effects in another aspect [42]. In individuals with a suitable health status prior to ART-initiation, the mentioned side-effects may decline the benefits [43]. Experiencing few ART-related side-effects associated to normal physical and mental health [43]. One of the examples of side-effects to be mentioned is sexual dysfunction which has a major effect on HRQoL even thogh frequently seen without iatrogenic reason [44].

Previous studies mostly demonstrated the a positive impact of ART on HRQoL. The reason may be because of the frequent enhancements in ART causing to lower side-effects and to likehoods of compound primary actions [45, 46]. ART treatment was autonomously related to a better physical health status [47] and suspension of ART treatment was related with lower mental health status [48]. Several studies indicated no HRQoL-differences between ART-treated and non-treated [4, 49]. The reason may be due to the fact that non-treated PLHIV had a more suitable health, which the treatment was not essential [49].

Given that this study measured CD4 count at less than 200, our findings indicate advanced HIV level of infection progression and its negative impacts on QoL. Usually individuals with this low of a CD4 count are diagnosed with HIV/AIDS. At this stage, CD4 count at lees than 200, the infection manifests the obvious demonstrations. The Asymptomatic stage of the infection convert to symptomatic stage (in stage 4 of the infection with physicial and observable demonstrtions), and according to WHO’s HIV/AIDS infection staging, we can observe the process of conversion of the HIV infection to AIDS as an observable illness. Therfore at this level of CD4 count, we can realize the infection as a disease [38].

However, in a study the results showed that there is no a significant difference between two ART and ART-naïve groups regarding to their counts of CD4 and the level of progression of infection [37]. In another review study the results presented the positive impact of higher level of CD4 counts on QoL, but the researcher stated this association may be not independent. They also concluded that adherence to ART treatment for more than 12 months may lead to better QoL and starting ART in patients with lower counts of CD4 cause better outcomes [50]. Irregardless, those with a CD4 count under 200 need greater attention by receiving appropriate palliative and supportive care.

In the other words, the previous research confirmed the association between immunological status and HRQoL: a higher CD4-cell count proved a better physical health [51–53]. Also it could estimate better physical health scores on follow-up measurements [54].

In patients who began ART at CD4 < 200 cells/μl thephysicalhealth status enhanced in comparison with patients with 200–350 cells/μl or > 350 cells/μl, the reason was that their physical health at the start point was worse [55]. In spite of that the present guidelines suggest to early beginning of ART (i.e. 350–500 celles/μl), because there it is proved that this findings in decreased progression to AIDS and decreased mortality [50].

According to another study the count of CD4 had negative association with HRQoL at the beginning, and after 12 months turned to positive [56]. One reason may be that PLHIV with a high CD4-count at the start tend to return to their good health and hence pondered their actual health status as not satisfying. After a while, PLHIV expanded other understanding and expectations about HRQoL and could think of their health status satisfying [56].

Other three researches suggested more relationship with mental health. A lower counts of CD4 cell was related with a lower mental health score (cross-sectional and at 12 months) [57, 58]. The reason may be because of the faster disease development in PLHIV with low CD4-counts, cauing to distress [57].

Considering the stage of the disease, a transparent influence on physical health has been determined: by progressing the stages of HIV physical health decreases [58, 59]. The lowest level of physical health has been in people with AIDS which it affects the future physical health in follow-up researches negatively [57, 60].

Of course in this stage the patients need to be considered by medcial practitioners and the ART should be continue with a more focus on the other co-morbidities and diseases that are suffering the patients. Controling the oral and esophageal infections, the Tuberclosis condition, sarcoma and other co-incident illness are very crucial in manging the pateints at this stage. This therapeutic plan may have a grat contribution in helping the patients and manging their health status and subsequently their QoL [61].

One of the challenging aspects in managing and controlling HIV among diagnosed population are co-morbidity and opportunistic infections. These infections may lead to enhanced health problems for patients and thus impact their QoL. However, our meta-analysis did not find a significant association between QoL and co-infection. This is supported by other studies that found no significant difference in QoL between mono-infected HIV/AIDS patients and those infected with hepatitis. In Iran, the results of a study showed HCV/HIV co-infected people have higher scores for physical domain but the HIV mono-infected individuals have had greater scores in social support and physical functioning [62].

Additionally, our study found a positive association between the time of diagnosis and accessibility of services on QoL among PWLHs. Diagnosing HIV/AIDS in its earliest stage and then designing in-time therapy with patients may have a positive impact on managing and controlling the illness. This can lead to a better QoL for PWLHs’ by preparing them useful coping strategies and improved resilience. One study in Estonia found that the QoL of among HIV/AIDS infected individuals that were diagnosed 12 months ago or more was higher than those who were diagnosed less than 12 months ago [7].

However, the influences in mental health were different. A longer time because the diagnosis was related with both lower [49] and higher mental health [63]. Lower mental health during the time may emerge in individuals where longitudinal follow-up caused to a successful control of HIV [49]. After that, PLHIV encountered difficulties to state mental problems because they were anticipated to feel happy because of their suitable health status. On the other words, a longer period may make the growth of effective coping approaches easier which may improve mental health [56]. It is essential to control mental health frequently by health care providers, regardless of the physical condition of the patient.

This study had several limtations. The included studies examined different covariations and had varying approaches to creating their models including logistic and linear models as well as some conducting univariate and other multivariate approaches. Examples of covariate classifications that differed from each study were education and income levels. Thus we stipulated the inclusion criteria indicated in method to allow for aggregation of data and to perform the meta-analysis.

## Conclusion

The results illustrated that time diagnosing and availability to hospital services had significant relationship with a higher QoL and CD4 < 200 was associated with a lower QoL. Providing comprehensive diagnostic and therapeutic facilities for early detection well as monitoring the health status of PWLHs’ can be a useful agenda to for policymakers who are looking to for managing HIV/AIDS infection and improve QoL of life in their community. Considering the medical needs of HIV/AIDS patients, developing supportive and therapeutic actions regarding their illness are recommended.

## Data Availability

The datasets used and/or analyzed during the current study are available from the corresponding author on reasonable request.

## Abbreviations

ART: Antiretroviral therapy
cART: Combination antiretroviral treatment
HAART: Highly Active Antiretroviral Therapy
HIV: Human Immunodeficiency Virus
HRQoL: health-related quality of life
ORs: Odds ratios
PRISMA: Preferred Reporting Items for Systematic Reviews and Meta-Analyses
PWLHs’: People Who Live with HIV/AIDS
QoL: Quality of Life
UNAIDS: United Nations joint program on HIV and AIDS

## References

[1] Flannelly LT, Inouye J (2001). Relationships of religion, health status, and socioeconomic status to the quality of life of individuals who are HIV positive. Issues in mental health nursing.

[2] Perez IR, Bano JR, Ruz ML, del Arco JA, Prados MC, Liaño JP, Rico PM, De la Torre LJ, Pardal JP, Gomez ML (2005). Health-related quality of life of patients with HIV: impact of sociodemographic, clinical and psychosocial factors. Qual Life Res.

[3] Egger M, Smith GD, Schneider M, Minder C (1997). Bias in meta-analysis detected by a simple, graphical test. Bmj.

[4] Briongos Figuero L, Bachiller Luque P, Palacios Martín T, González Sagrado M, Eiros Bouza J (2011). Assessment of factors influencing health-related quality of life in HIV-infected patients. HIV Med.

[5] UNAIDS The Joint United Nations Programme on HIV/AIDS [https://www.unaids.org/en/keywords/unaids-joint-united-nations-programme-hivaids]. 12349391

[6] Thapa R, Amatya A, Pahari DP, Bam K, Newman MS (2015). Nutritional status and its association with quality of life among people living with HIV attending public anti-retroviral therapy sites of Kathmandu Valley, Nepal. AIDS Res Ther.

[7] Rüütel K, Pisarev H, Loit H-M, Uusküla A (2009). Factors influencing quality of life of people living with HIV in Estonia: a cross-sectional survey. J Int AIDS Soc.

[8] Mwesiga EK, Mugenyi L, Nakasujja N, Moore S, Kaddumukasa M, Sajatovic M (2015). Depression with pain co morbidity effect on quality of life among HIV positive patients in Uganda: a cross sectional study. Health Qual Life Outcomes.

[9] Xie F, Zheng H, Huang L, Yuan Z, Lu Y (2019). Social capital associated with quality of life among people living with HIV/AIDS in Nanchang, China. Int J Environ Res Public Health.

[10] Rueda S, Raboud J, Mustard C, Bayoumi A, Lavis JN, Rourke SB (2011). Employment status is associated with both physical and mental health quality of life in people living with HIV. AIDS Care.

[11] Abrefa-Gyan T, Cornelius LJ, Okundaye J (2016). Socio-demographic factors, social support, quality of life, and HIV/AIDS in Ghana. J Evid Inf Soc Work.

[12] Ghisvand H, Higgs P, Noroozi M, Ghaedamini Harouni G, Hemmat M, Ahounbar E, Haroni J, Naghdi S, Nazeri Astaneh A, Armoon B. Social and demographical determinants of quality of life in people who live with HIV/AIDS infection: evidence from a meta-analysis. Biodemography Soc Biol. 2019:1–16. 10.1080/19485565.2019.1587287 30882251

[13] Bhatta DN, Liabsuetrakul T, McNeil EB (2017). Social and behavioral interventions for improving quality of life of HIV infected people receiving antiretroviral therapy: a systematic review and meta-analysis. Health Qual Life Outcomes.

[14] Miners A, Phillips A, Kreif N, Rodger A, Speakman A, Fisher M, Anderson J, Collins S, Hart G, Sherr L (2014). Health-related quality-of-life of people with HIV in the era of combination antiretroviral treatment: a cross-sectional comparison with the general population. Lancet HIV.

[15] Crum NF, Riffenburgh RH, Wegner S, Agan BK, Tasker SA, Spooner KM, Armstrong AW, Fraser S, Wallace MR (2006). Comparisons of causes of death and mortality rates among HIV-infected persons: analysis of the pre-, early, and late HAART (highly active antiretroviral therapy) eras. J Acquir Immune Defic Syndr.

[16] Rodriguez-Penney AT, Iudicello JE, Riggs PK, Doyle K, Ellis RJ, Letendre SL, Grant I, Woods, the HIV neurobehavioral research program group SP: **C**o-morbidities in persons infected with HIV: increased burden with older age and negative effects on health-related quality of life. AIDS Patient Care STDs 2013, 27(1):5–16. 10.1089/apc.2012.0329 PMC3545369 23305257

[17] Emuren L, Welles S, Evans AA, Polansky M, Okulicz JF, Macalino G, Agan BK, Group IDCRPHW (2017). Health-related quality of life among military HIV patients on antiretroviral therapy. PLoS One.

[18] Abebe Weldsilase Y, Likka MH, Wakayo T, Gerbaba M. Health-related quality of life and associated factors among women on antiretroviral therapy in health facilities of Jimma town Southwest Ethiopia. Adv Public Health. 2018;2018.

[19] Perez IR, Bano JR, Ruz ML, del Arco JA, Prados MC, Liano JP, Rico PM, De la Torre LJ, Pardal JP, Gomez ML (2005). Health-related quality of life of patients with HIV: impact of sociodemographic, clinical and psychosocial factors. Qual Life Res.

[20] Ruiz-Pérez I, de Labry-Lima AO, López-Ruz MÁ, del Arco-Jiménez A, Rodríguez-Baño J, Causse-Prados M, Pasquau-Liaño J, Martín-Rico P, Prada-Pardal JL, de la Torre-Lima J (2005). Estado clínico, adherencia al TARGA y calidad de vida en pacientes con infección por el VIH tratados con antirretrovirales. Enfermedades infecciosas y microbiologia clinica.

[21] Jia H, Uphold CR, Wu S, Chen GJ, Duncan PW (2005). Predictors of changes in health-related quality of life among men with HIV infection in the HAART era. AIDS Patient Care STDs.

[22] Campos LN, César CC, Guimarães MDC (2009). Quality of life among HIV-infected patients in Brazil after initiation of treatment. Clinics.

[23] Shamseer L, Moher D, Clarke M, Ghersi D, Liberati A, Petticrew M, Shekelle P, Stewart LA (2015). Preferred reporting items for systematic review and meta-analysis protocols (PRISMA-P) 2015: elaboration and explanation. Bmj.

[24] Stroup DF, Berlin JA, Morton SC, Olkin I, Williamson GD, Rennie D, Moher D, Becker BJ, Sipe TA, Thacker SB (2000). Meta-analysis of observational studies in epidemiology: a proposal for reporting. Jama.

[25] Moradi-Joo M, Ghiasvand H, Noroozi M, Armoon B, Noroozi A, Karimy M, Rostami A, Mirzaee MS, Hemmat M (2019). Prevalence of skin and soft tissue infections and its related high-risk behaviors among people who inject drugs: a systematic review and meta-analysis. J Subst Abus.

[26] Stang A (2010). Critical evaluation of the Newcastle-Ottawa scale for the assessment of the quality of nonrandomized studies in meta-analyses. Eur J Epidemiol.

[27] Higgins JP, Green S: Cochrane handbook for systematic reviews of interventions, vol. 4: John Wiley & Sons; 2011.

[28] Begg CB, Mazumdar M. Operating characteristics of a rank correlation test for publication bias. Biometrics. 1994:1088–101. 7786990

[29] Aragonés-López C, Pérez-Ávila J, Smith Fawzi MC, Castro A (2012). Quality of life of people with HIV/AIDS receiving antiretroviral therapy in Cuba: a cross-sectional study of the national population. Am J Public Health.

[30] Deribew A, Deribe K, Reda AA, Tesfaye M, Hailmichael Y, Maja T, Colebunders R (2013). Change in quality of life: a follow up study among patients with HIV infection with and without TB in Ethiopia. BMC Public Health.

[31] George S, Bergin C, Clarke S, Courtney G, Codd MB (2016). Health-related quality of life and associated factors in people with HIV: an Irish cohort study. Health Qual Life Outcomes.

[32] Tesfay A, Gebremariam A, Gerbaba M, Abrha H. Gender differences in health related quality of life among people living with HIV on highly active antiretroviral therapy in Mekelle town Northern Ethiopia. BioMed Res Int. 2015;2015. 10.1155/2015/516369 PMC4303010 25632393

[33] Boyer S, Protopopescu C, Marcellin F, Carrieri MP, Koulla-Shiro S, Moatti J-P, Spire B, Group ES (2011). Performance of HIV care decentralization from the patient’s perspective: health-related quality of life and perceived quality of services in Cameroon. Health Policy Plan.

[34] Lan G-L, Yuan Z-K, Clements-Nolle KD, Cook A, Yuan L-L, Xu Q-Y, Jiang H-Y, Zheng H-L, Wang L, Yang W (2016). Social capital and quality of life among people living with HIV/AIDS in Southeast China. Asia Pacific J Public Health.

[35] Friend-du Preez N, Peltzer K (2010). HIV symptoms and health-related quality of life prior to initiation of HAART in a sample of HIV-positive south Africans. AIDS Behav.

[36] Louwagie GM, Bachmann MO, Meyer K, le R Booysen F, Fairall LR, Heunis C: Highly active antiretroviral treatment and health related quality of life in south African adults with human immunodeficiency virus infection: a cross-sectional analytical study. BMC Public Health 2007, 7(1):244. 10.1186/1471-2458-7-244 PMC2194770 17854510

[37] Nglazi MD, West SJ, Dave JA, Levitt NS, Lambert EV (2014). Quality of life in individuals living with HIV/AIDS attending a public sector antiretroviral service in Cape Town South Africa. BMC Public Health.

[38] Organization WH (2016). Consolidated guidelines on the use of antiretroviral drugs for treating and preventing HIV infection: recommendations for a public health approach: World Health Organization.

[39] Basavaraj K, Navya M, Rashmi R (2010). Quality of life in HIV/AIDS. Indian J Sex Transm Dis AIDS.

[40] Mutabazi-Mwesigire D, Katamba A, Martin F, Seeley J, Wu AW (2015). Factors that affect quality of life among people living with HIV attending an urban clinic in Uganda: a cohort study. PLoS One.

[41] Liping M, Peng X, Haijiang L, Lahong J, Fan L (2015). Quality of life of people living with HIV/AIDS: a cross-sectional study in Zhejiang province China. PloS one.

[42] Liu C, Ostrow D, Detels R, Hu Z, Johnson L, Kingsley L, Jacobson LP (2006). Impacts of HIV infection and HAART use on quality of life. Qual Life Res.

[43] Preau M, Protopopescu C, Spire B, Sobel A, Dellamonica P, Moatti JP, Carrieri MP (2007). Health related quality of life among both current and former injection drug users who are HIV-infected. Drug Alcohol Depend.

[44] Koole O, Noestlinger C, Colebunders R (2007). Quality of life in HIV clinical trials: why sexual health must not be ignored. PLoS Clin Tials.

[45] Este JA, Cihlar T (2010). Current status and challenges of antiretroviral research and therapy. Antivir Res.

[46] Airoldi M, Zaccarelli M, Bisi L, Bini T, Antinori A, Mussini C, Bai F, Orofino G, Sighinolfi L, Gori A (2010). One-pill once-a-day HAART: a simplification strategy that improves adherence and quality of life of HIV-infected subjects. Patient Prefer Adherence.

[47] Fleming CA, Christiansen D, Nunes D, Heeren T, Thornton D, Horsburgh CR, Koziel MJ, Graham C, Craven DE (2004). Health-related quality of life of patients with HIV disease: impact of hepatitis C coinfection. Clin Infect Dis.

[48] Liu C, Johnson L, Ostrow D, Silvestre A, Visscher B, Jacobson LP (2006). Predictors for lower quality of life in the HAART era among HIV-infected men. J Acquir Immune Defic Syndr.

[49] Zinkernagel C, Taffe P, Rickenbach M, Amiet R, Ledergerber B, Volkart AC, Rauchfleisch U, Kiss A, Werder V, Vernazza P (2001). Importance of mental health assessment in HIV-infected outpatients. J Acquir Immune Defic Syndr.

[50] Degroote S, Vogelaers D, Vandijck DM (2014). What determines health-related quality of life among people living with HIV: an updated review of the literature. Arch Public Health.

[51] Murri R, Fantoni M, Del Borgo C, Visona R, Barracco A, Zambelli A, Testa L, Orchi N, Tozzi V, Bosco O (2003). Determinants of health-related quality of life in HIV-infected patients. AIDS Care.

[52] Armon C, Lichtenstein K (2012). The associations among coping, nadir CD4+ T-cell count, and non-HIV-related variables with health-related quality of life among an ambulatory HIV-positive patient population. Qual Life Res.

[53] Call SA, Klapow JC, Stewart KE, Westfall AO, Mallinger AP, DeMasi RA, Centor R, Saag MS (2000). Health-related quality of life and virologic outcomes in an HIV clinic. Qual Life Res.

[54] Lorenz KA, Cunningham WE, Spritzer KL, Hays RD (2006). Changes in symptoms and health-related quality of life in a nationally representative sample of adults in treatment for HIV. Qual Life Res.

[55] Nieuwkerk PT, Hillebrand-Haverkort ME, Vriesendorp R, Frissen PJ, de Wolf F, Sprangers MA, Group AS (2007). Quality of life after starting highly active antiretroviral therapy for chronic HIV-1 infection at different CD4 cell counts. J Acquir Immune Defic Syndr.

[56] Jia H, Uphold CR, Zheng Y, Wu S, Chen GJ, Findley K, Duncan PW (2007). A further investigation of health-related quality of life over time among men with HIV infection in the HAART era. Qual Life Res.

[57] Protopopescu C, Marcellin F, Spire B, Préau M, Verdon R, Peyramond D, Raffi F, Chêne G, Leport C, Carrieri M-P (2007). Health-related quality of life in HIV-1-infected patients on HAART: a five-years longitudinal analysis accounting for dropout in the APROCO-COPILOTE cohort (ANRS CO-8). Qual Life Res.

[58] Préau M, Marcellin F, Carrieri MP, Lert F, Obadia Y, Spire B, Group VS: Health-related quality of life in French people living with HIV in 2003: results from the national ANRS-EN12-VESPA study. In: LWW; 2007. 10.1097/01.aids.0000255081.24105.d7 17159583

[59] Hays RD, Cunningham WE, Sherbourne CD, Wilson IB, Wu AW, Cleary PD, McCaffrey DF, Fleishman JA, Crystal S, Collins R (2000). Health-related quality of life in patients with human immunodeficiency virus infection in the United States: results from the HIV cost and services utilization study. Am J Med.

[60] Préau M, Vincent E, Spire B, Reliquet V, Fournier I, Michelet C, Leport C, Morin M, Group AS (2005). Health-related quality of life and health locus of control beliefs among HIV-infected treated patients. J Psychosom Res.

[61] Owiti PO, Penner J, Oyanga A, Huchko M, Onchiri FM, Cohen C, Bukusi EA: WHO stage 4 conditions among adults accessing outpatient HIV care: A retrospective cohort study in Kisumu, Kenya. J Acq Imm Def Syndr (1999) 2014, 65(4):e152. 10.1097/QAI.0000000000000020 PMC3939608 24577188

[62] Sabouri S, Delavar A, Jabbari H (2016). Quality of life among human immunodeficiency virus-1 infected and human immunodeficiency virus-1/hepatitis C virus co-infected individuals in Iranian patients. Nigerian medical journal: journal of the Nigeria Medical Association.

[63] Rueda S, Raboud J, Mustard C, Bayoumi A, Lavis JN, Rourke SB (2011). Employment status is associated with both physical and mental health quality of life in people living with HIV. AIDS Care.

